# Effects of type and distribution of clay minerals on the physico-chemical and geomechanical properties of engineered porous rocks

**DOI:** 10.1038/s41598-023-33103-4

**Published:** 2023-04-10

**Authors:** Soha Iranfar, Mohammad Mehdi Karbala, Mahmood Shakiba, Mohammad Hossein Shahsavari

**Affiliations:** 1 Department of Petroleum Engineering, Abadan Faculty of Petroleum, Petroleum University of Technology (PUT), Abadan, Iran; 2 Rock Mechanics Division, School of Engineering, Tarbiat Modares University, Tehran, Iran; 3 Department of Chemical Engineering, Faculty of Engineering, Ferdowsi University of Mashhad, Mashhad, Iran; 4 Department of Petroleum Engineering, Amirkabir University of Technology (Tehran Polytechnic), Tehran, Iran

**Keywords:** Geochemistry, Mineralogy, Petrology

## Abstract

The study of the properties of engineered rocks is of great importance to researchers in engineering sciences such as petroleum, mining, and civil engineering owing to their wide application in these fields. In the present study, a physico-chemical and geomechanical investigation was carried out on the effects of different clay minerals on porous rocks. Various chemical products formed during chemical interactions between cement, clay minerals, and water can change the pore structure and thus the rock characteristics. The results of the current study showed that increasing the clay content could remarkably reduce the porosity and permeability of the rock by an average of 86% and 6.76%, respectively. In this regard, samples containing kaolinite were further influenced due to their new pore structure. Moreover, a power relationship was found between sonic velocity and porosity, which can be used to predict rock properties. Chemical analysis indicated an amplification in quantities of chemical products, particularly calcium silicate hydrate and portlandite, due to an increase in clay content. The impacts of porosity and cementation quality as two main factors on rock strength have also been studied. The outcomes revealed that a reduction in porosity could compensate for detrimental effects of poor bond quality and consequently improved UCS by up to 30% in samples containing kaolinite, while decreasing the degree of cementation prevailed over the porosity reduction in specimens including illite and resulted in a 14% decrease in UCS. The effects of porosity and bond quality on UCS would cancel each other out in samples containing bentonite. It is worth noting that when it comes to changes in geomechanical characteristics, the dominant factor (i.e., porosity reduction or cementation quality) determines the ultimate effect of clay minerals on the properties of engineered porous rocks.

## Introduction

Accurate knowledge of engineered cemented porous media is of particular importance in geology, petroleum, and civil engineering^1,2^. Artificial cemented porous media are usually made using two materials including aggregates and binder cement. Different types of binders include Portland cement, geopolymer, sintered materials, and precipitated calcite^3–7^. However, Portland cement binders have the most advantages over other methods in terms of geological and mineralogical similarity to real sandstones^8^. In addition, such method offers high potential for reproducibility, low anisotropy, and controllable physico-chemical and geomechanical properties^9^.

Many studies investigated the effects of water content, curing time, porosity, moisture, particle size distribution (PSD), amount and type of cement on the uniaxial compressive strength (UCS) of synthetic sandstones^2,10–17^. In addition, the results of such studies can be used in space concrete technology because the PSD ranges from 0 to 1 mm are the same for synthetic sandstones and Martian and Lunar regoliths^18,19^.

One of the important parameters that has been less investigated is the clay content in the structure of synthetic porous media^20^. These minerals have a significantly impact on the properties of porous media, therefore it is worthwhile to understand their effect on both technical industry and scientific research^21^. Such materials are characterized by strong adsorption capacity, large surface area and high swelling capacity^22–25^. These minerals are a subgroup of anisotropic phyllosilicates (one of the main groups of aluminosilicates) containing an octahedral (O) alumina and one or two tetrahedral (T) silica sheets, which together form a basic unit consisting of either 2:1 tetrahedral-octahedral-tetrahedral (TOT) or 1:1 tetrahedral-octahedral (TO) layers^26,27^. The physical and chemical characteristics of a particular clay mineral depend largely on its structure (i.e. the arrangement and composition of the octahedral and tetrahedral sheets), cation and anion exchange capacity, specific surface area, and adsorption ability^27,28^. Ions can be replaced in a wide range in response to the chemical properties of the original and surrounding environment within the crystal structure of a single clay mineral species^29^. Illite, kaolinite, and bentonite are the most abundant clays in sandstones^30–32^. Illite ((K, H_3_O)(Al, Mg, Fe)_2_(Si, Al)_4_O_10_[(OH)_2_, (H_2_O)]), the most frequent primary clay mineral in marine shales with a 2:1 structure, is of a high cation exchange capacity that allows it to absorb and retain much water. Its structure consists of silicate sheets that can expand upon contact with water, resulting in volume increase and swelling^29,33–36^. In addition, it has a structure like montmorillonite, but potassium ions bind its layers together^27,37^. Bentonite ((Na, Ca)_0.33_(Al, Mg)_2_Si_4_O_10_(OH)_2_.n(H_2_O)) is a ubiquitous clay derived from two different mineral forms, including sodium bentonite containing sodium as an exchangeable ion (swelling), and calcium bentonite with two water layers containing calcium as an exchangeable ion (non-swelling). Bentonite can absorb a great deal of water by increasing its volume by 12 to 15 times. However, its swelling capacity is moderate compared to illite due to the presence of other minerals such as quartz and feldspar^38–40^. Kaolinite (Al_2_O_3_∙2SiO_2_∙2H_2_O) is part of the kaolin-serpentine group with low cation exchange capacity and packed layers of a tetrahedral silica sheet and an octahedral alumina sheet (1:1 layer type of clay), which makes it less expansive when exposed to water and thus less swelling than illite or bentonite^35,41–43^.ꞏ

Clays and cement are particle materials with high reactivity interacting with water^44^. The clay fabric and cementation process control the strength and deformation properties of the clay^45^. The strength (i.e. UCS) of the resulting clay–cement mixture is mainly determined by the cement content, water-cement ratio, and the curing conditions and follows linear relationships at various curing times^46–50^. Understanding chemical interaction between clay soils and water can be useful in making such systems suitable for engineering purposes such as materials science, petroleum engineering, geology, geophysics, sedimentology, and environmental science^51^. The main topics of clay mineral–water interaction research include hydration and dehydration of water, swelling of clay minerals, aggregate assembly, and particle arrangements of clay minerals in an aqueous solution^52^. These properties define the optimal conditions for a well-dispersed system as well as the coagulation, flotation, and dispersion characteristics in suspension systems. Clay minerals adsorb water molecules and hydrated cations in aqueous solutions, leading to hydration on their external and internal surfaces^53,54^.

To date, several researchers studied clay minerals’ effects on the physical and chemical characteristics of porous media and concretes^55^. Han^56^ carried out some experimental tests to measure the compressional and shear wave velocities and porosity in samples with 0 to 30% cement content^56^. His findings suggest that clay particles can undergo plastic deformation, and higher clay content results in porosity reduction and velocities increment. In addition, in comparison to pure sand grains, saturated clay showed a much lower shear velocity. Horpibulsuk et al. analyzed the compressibility characteristics of cement-admixed clay^45,57^. It was concluded that cement content is the main effective parameter governing the stress–strain curve at the post-yield state. Furthermore, Horpibulsuk et al. showed that the strength enhancement in the blended cement which stabilized clay is governed by cementitious products due to the combined effect of dispersion and hydration^58^. Also, experimental tests indicated that reducing the water-clay/cement ratio (wc/c) results in higher cementation bond strength and yield stress^59–61^. The mixtures of cement, sand, and kaolin were investigated by Khelifi et al. to design extruded building materials with less environmental effect^61^. The mechanical testing results indicate that using cement, sand, and kaolin mixtures can generate remarkable compressive strength. Aksu et al. carried out a series of permeability tests on unconsolidated specimens with varying clay content^41^. They combined coreflood experiments with X-ray µ-computed tomography (µ-CT) to study the clay minerals swelling and its effect on the permeability of unconsolidated porous media. They found that montmorillonite and kaolinite clay minerals could reduce permeability^41^. Supandi et al. studied the effects of illite and kaolinite on mechanical and physical properties of a rock such as stress, strain, cohesion, friction angle, void ratio, natural water content, and wet density of claystone^62^. The results indicated that illite content negatively affected mechanical properties and increased natural moisture content, wet density, and void ratio, while kaolinite did not have a notable impact on such properties.

According to literature, the effects of type, content and distribution of clay minerals without swelling phenomenon on the physical, chemical, and geomechanical properties of engineered porous rocks, have not been explicitly investigated. For this purpose, bentonite (with a 2:1 (TOT) structure), kaolinite (with a 1:1 (TO) structure), and illite, which has a 2:1 (TOT) structure were applied in this study at two weight percentages (i.e. 5% and 10%) to prepare engineered samples. The results of this study can help civil and petroleum engineers know the behavior of engineered rocks for a variety of utilization. Moreover, geotechnical engineering as a branch of civil engineering that deals with the principles of soil and rock mechanics (i.e. porosity, permeability, and compressive strength as important properties of soils) and has various applications in the mining and petroleum engineering can use the results of the current study to analyze site conditions and design earthworks, retaining structures, and foundations. The more information available on the relationships between rock and soil properties, the better the behavior of the materials can be determined, and thus an accurate prediction of their future performance can be made.

## Sample preparation

### Sample composition

In order to determine the influence of different clays on the physico-chemical and geomechanical characteristics of engineered porous rocks, six series of samples containing kaolinite, illite, and bentonite with two levels of clay content (5% and 10%) were prepared according to the ASTM C305 standard^6^. It is worth mentioning that sand particle sizes remained constant in the range of 0.1 to 0.8 mm (i.e. very fine to coarse sand particles) with a density of 2.64 gr/cm^3^. Moreover, Portland cement type II was used as a binder between the particles with a density of 3.11 gr/cm^3^. Table 1 shows the X-ray fluorescence spectrometry (XRF) analysis of the cement, sand particles, ans clay minerals applied here.

**Table 1:** XRF analysis of cement, sand grains, and clays used for preparing core sample (%).

Chemical composition	SiO_2_	Al_2_O_3_	BaO	CaO	Fe_2_O_3_	K_2_O	MgO	MnO	Na_2_O	P_2_O_5_	SO_3_	TiO_2_	LOI
Cement	20.53	4.27	0.05	63.43	3.21	0.71	2.85	0.16	0.35	0.05	2.51	0.31	1.57
Sand	79.86	7.44	0.08	3.96	0.44	2.93	0.25	0.14	1.78	–	1.32	0.06	1.49
Kaolinite	59.86	19.21	–	1.54	5.28	2.26	2.53	0.07	1.16	0.12	–	1.14	6.83
Illite	61.48	17.84	–	1.73	5.31	5.65	2.74	–	0.31	–	0.22	–	4.72
Bentonite	66.25	16.73	–	2.56	4.22	2.38	1.66	0.15	1.44	0.14	–	0.72	3.75

To provide artificially-made core samples in this study, a composition proposed by Shakiba et al. was used^6,63^. The composition consists of sand, cement, water and a clay mineral according to the results of porosity, permeability and compressive strength tests^6^. They first proposed nine compositions for synthetic sandstones prepared based on the ASTM C305 method, and eventually, the composition including 12 wt% water, 11 wt% cement, 5 wt% clay mineral, and 72 wt% sand was considered to be an appropriate composition for artificial sandstones^6,63^. In the current research, a decrease in the amount of sand (up to 5 wt%) was due to an increase in the clay content of samples K10, I10, and B10.

The specific surface area of a material is an important factor in many applications such as adsorption, mineral dissolution, filtration, and precipitation, and understanding its properties is crucial to the design and optimization of processes using these materials^64,65^. A larger specific surface area generally means a higher capacity for adsorption and chemical reactions^65^. In terms of specific surface area, kaolinite has a relatively low value compared to illite and bentonite. This is due to the relatively flat and smooth surface of kaolinite particles, which limits the surface area available for adsorption and chemical reactions^66^. In contrast, illite and bentonite have a much larger specific surface area due to their more complex crystal structures and a greater degree of surface roughness^64^. Table 2 shows the material composition of the specimens, the free swelling index (FSI), and the specific surface area of the clay minerals.

**Table 2:** Composition of core samples and FSI and specific surface area of clays.

Sample code	Water (wt%)	Cement (wt%)	Sand (wt%)	Clay (wt%)	Type of clay	FSI (%)	Specific surface area (m^2^/g)
K5	12	11	72	5	Kaolinite	35	20
K10	12	11	67	10	Kaolinite	35	20
I5	12	11	72	5	Illite	340	72
I10	12	11	67	10	Illite	340	72
B5	12	11	72	5	Bentonite	154	448
B10	12	11	67	10	Bentonite	154	448

### Curing process

**Figure 1:**
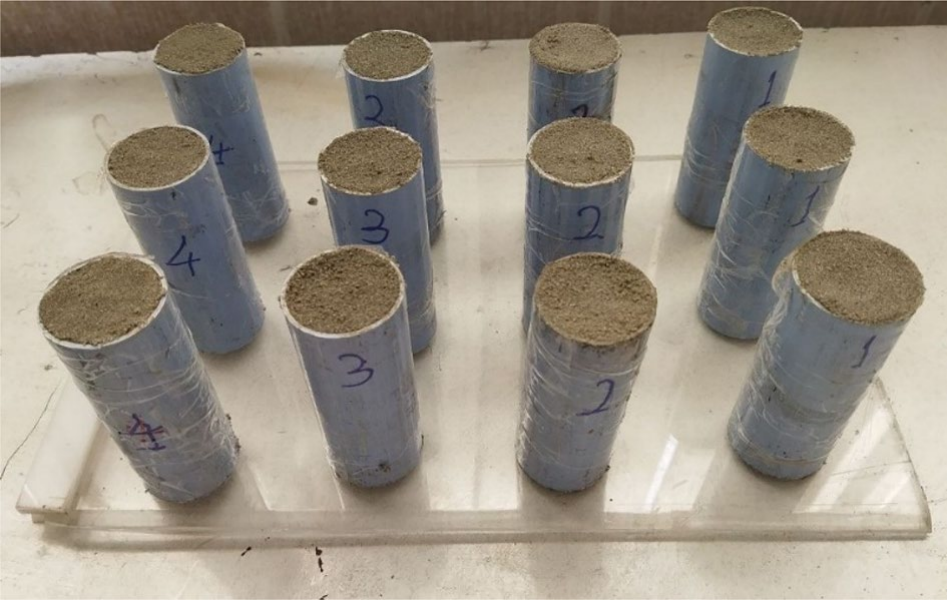
UPVC cylindrical molds and uncured samples.

Based on the ASTM C305 standard, after mixing the materials in a mixer and obtaining a homogeneous mortar, the paste is poured into several analogous molds for initial curring^6,12^. Several UPVC cylindrical containers with an inner diameter of 38 mm and a height of 100 mm, were used to make an initial mold for the samples. Figure 1 illustrates uncured specimens in the molds.

**Figure 2:**
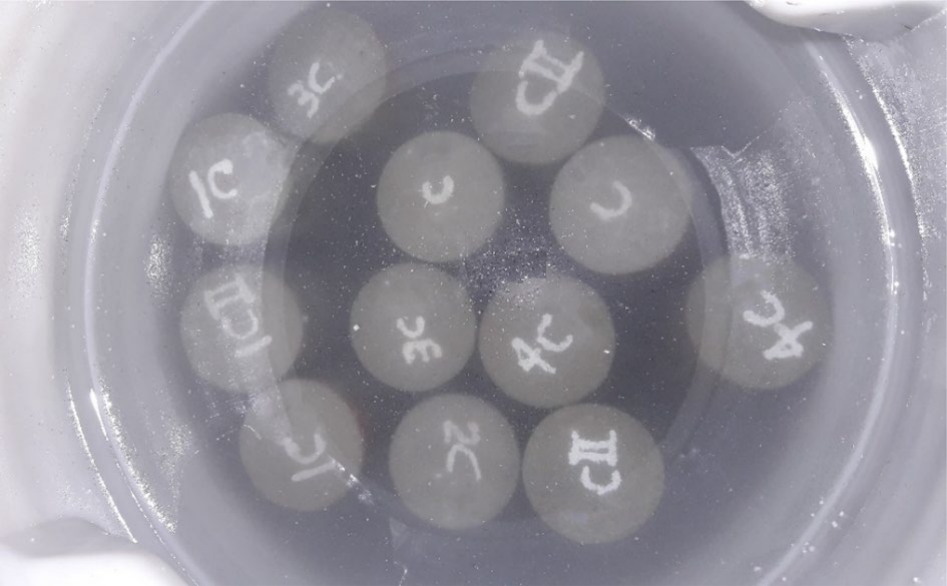
Bare core samples in the calcite solution.

All the cylindrical containers were placed on a vibrating plate to make a uniform texture for all the specimens without any cavity. Afterward, wet samples were placed in an absorption oven (primary curing) at a temperature of 22 °C for 24 h by remaining the moisture of the samples constant to give them a rigid form. All the molds were carefully removed at the end of this stage, and the final curing stage was initiated by placing the samples in the calcite solution for 28 days. A saturated calcite solution can enhance the quality of the cementation process and ensure that all the pore throats are interconnected^6^. Clay minerals typically have negatively charged surfaces that repel one another, and this repulsion causes swelling when the clay exposes to water. It is worth noting that the addition of salts to a solution containing clay minerals can prevent the clay from swelling, thus the swelling effect in the experiments would be negligible^67–70^. The positively charged ions in the salt can disrupt the electrostatic forces and neutralize some of the negative charges on the clay particles that cause the clay to swell in the presence of water. Under these conditions, the repulsive forces between the clay particles would reduce, preventing or reducing swelling. Various types of salts can be utilized for this purpose, including NaCl, CaCl_2_, KCl, and Na_2_SO_4_^67,71–73^. In this study, KCl was added to the mixing water used to prepare engineered specimens. Figure 2 shows the bare core samples in the calcite solution.

**Figure 3:**
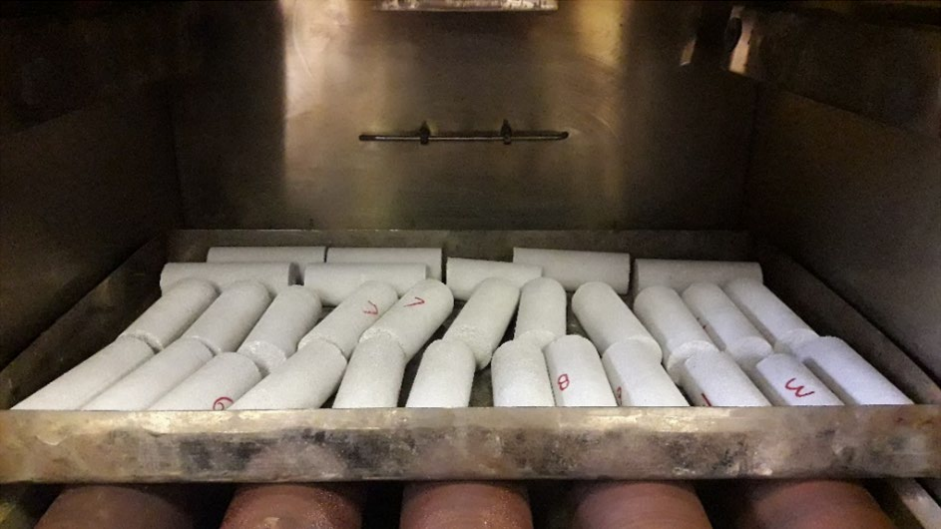
Dried samples in the oven.

After 28 days, core specimens were placed in an oven at 80 °C for 24 h to drain the remaining moisture entirely, as shown in Fig. 3. Finally, the dried samples were trimmed and flattened on both sides to prepare them for measuring the physical and geomechanical characteristics.

## Results and discussion

### Physical properties: porosity and permeability

**Figure 4:**
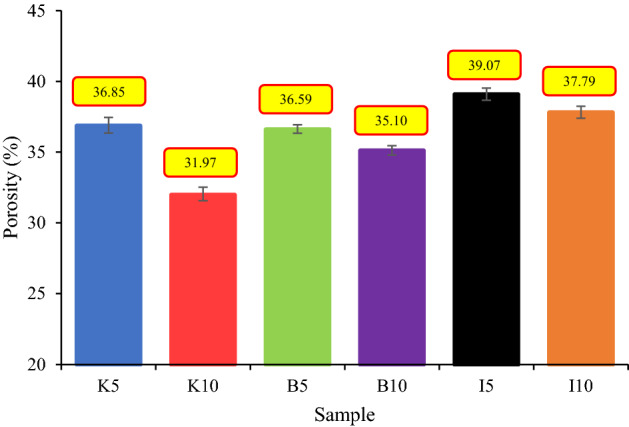
Porosity of samples.

**Figure 5:**
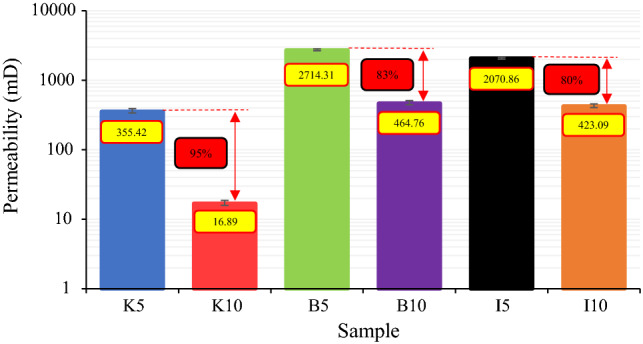
Permeability of all specimens.

Porosity and permeability, as two physical properties controlling fluid volume and fluid flow in rock, are of great importance in physico-chemical evaluation^15^. The porosity and permeability of dried samples were measured by gas permeameter and porosimeter. In this study, six series of synthetic cementing materials with two levels of clay content (5% and 10%) have been made to investigate the effects of various clay minerals on their physical and geomechanical characteristics. The dimensions and physical properties of samples are given in Table 3 and Figs. 4 and 5.

**Table 3:** Dimensions and clay content of samples.

Sample	Clay type	Diameter (cm)	Length (cm)	Clay content (wt%)
K5	Kaolinite	3.70	7.46	5
B5	Bentonite	3.70	7.35	5
I5	Illite	3.70	7.40	5
K10	Kaolinite	3.71	7.43	10
B10	Bentonite	3.70	7.32	10
I10	Illite	3.71	7.41	10

**Figure 6:**
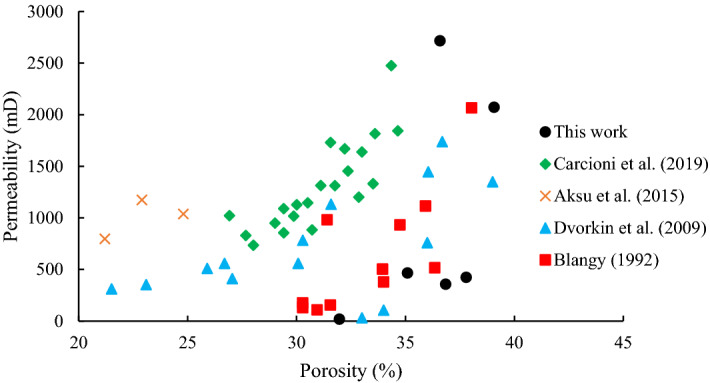
Comparison between rock porosity and permeability of this work and the literature (data from^41,74–76^).

As can be inferred from data in Table 3, an increase in clay content can severely affect permeability with little influence on porosity. In this regard, kaolinite drastically impacted these parameters as an increase of 5% in clay percentage led to a reduction of permeability and porosity up to 95% and 13%, respectively. The same change in clay content (i.e. 5%) in samples containing bentonite and illite resulted in an 83% and 80% reduction in permeability, respectively. According to Figs. 4 and 6, rock permeability is much more sensitive to composition than porosity, and consequently, it can be acutely influenced by any exterior elements^12^. To compare the rock porosity and permeability of the samples used in the current study with real rock specimens from the literature, all data are shown in Fig. 6.

It should be noted that all rock samples shown in Fig. 6 contain kaolinite, illite, bentonite, and smectite as clay minerals. As can be seen from Fig. 6, the synthetic samples used in this study have a high similarity to real sandstones, therefore they can largely mimic the conditions corresponding to real samples.

**Figure 7:**
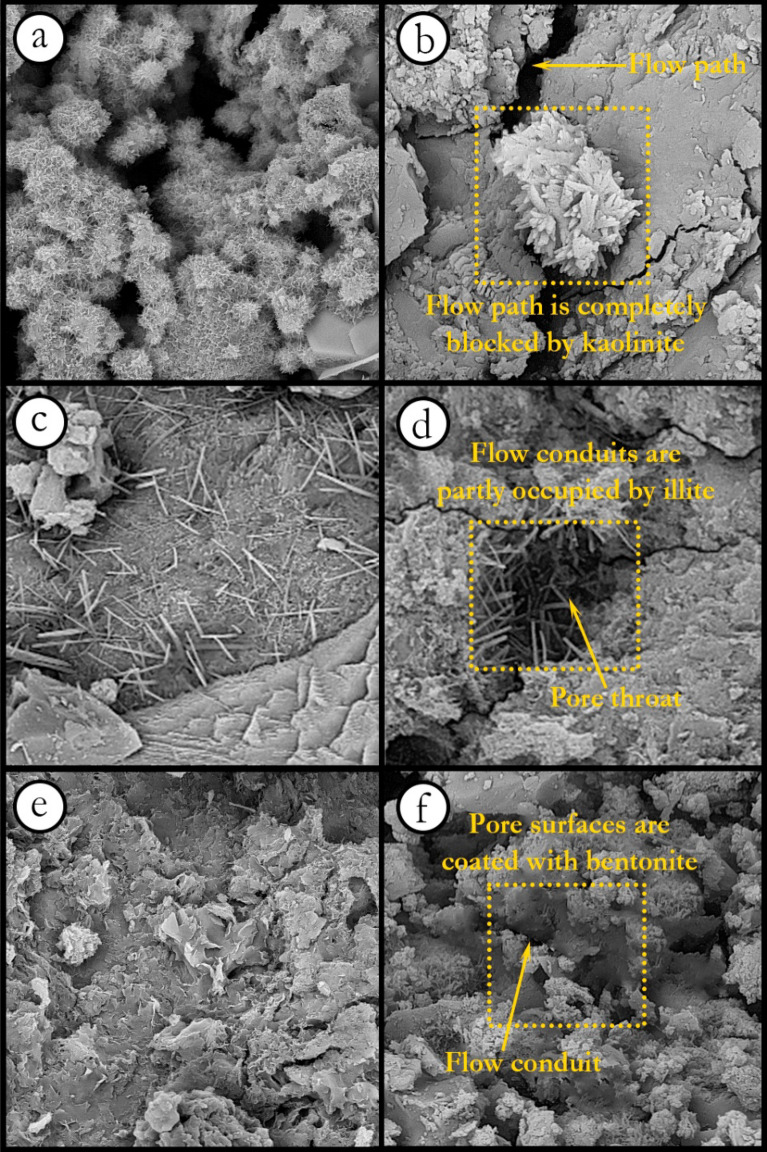
FESEM images of samples containing (**a**) kaolinite, (**c**) illite, (**e**) bentonite (view field: 28.9 µm), and magnified images showing fluid paths (**b**) kaolinite, (**d**) illite, and (**f**) bentonite (view field: 9.63 µm).

To find out the reasons behind such observations in porosity and permeability reduction, three magnified images were taken from thin slabs of samples using Field Emission Scanning Electron Microscopy (FESEM) performed on a TESCAN MIRA3 scanning electron microscope as shown in Fig. 7.

The product shape of reactions between clay minerals and cement in the aqueous solution can be observed in Fig. 7. Permeability as a physical property of a rock can be greatly affected by changes in rock texture and, consequently, flow paths^6,12,77^. As a result, an increase in clay content can dramatically reduce the permeability of the specimen by obstructing flow conduits and forming a new rock structure, as shown in Fig. 7b,d,e. From Fig. 7a, it can be seen that the structure of the sample containing kaolinite has a fluffy shape. The fluffy piles can completely block the connecting ways between the pores, as depicted in Fig. 7b. Accordingly, the permeability of the sample would be severely reduced. As can be observed in Fig. 7b, the chemical products in the illite-containing sample led to the formation of needle arrays. Under these conditions, although the pore structure and pore throats are affected by new elements (i.e. chemical products), the permeability of the specimen is less influenced than in the sample containing kaolinite. Bentonite can cover pore surfaces (i.e. sand particles) because of its high specific surface area, as demonstrated in Fig. 7e^78^. To put it in a nutshell, increasing the amount of unfavorable elements (i.e. clay content in this study) results in an adverse effect on fluid flow through the porous media.

**Figure 8:**
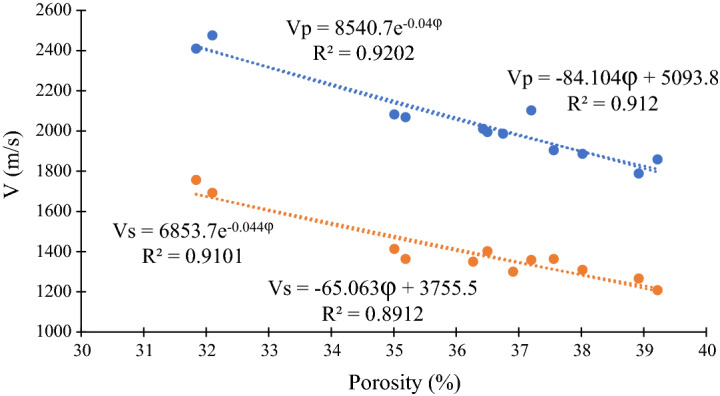
Exponential and linear relations between rock porosity and sonic velocity.

Many researchers have reported a linear or power relation between porosity and both compressive and shear sonic velocities^79–85^. In this study, SonicViewer SX-XP Model-5251C apparatus was used to measure ultrasonic P and S wave propagation. The results also indicated that there are both exponential and linear trends between porosity and sonic velocity, as depicted in Fig. 8.

As shown in Fig. 8, both compressive and shear sonic velocities demonstrate good exponential trends with porosity data, as their squared regression coefficient values, are 0.9202 and 0.9101, respectively. It is worth mentioning that the relation between sonic velocity and porosity not only concerns sample porosity but also is related to the type of porosity and pore geometry^79^. Although, the pore geometries differ among specimens, structurally uniform and identical structure in each sample can lessen the effects of pore geometry. This can be considered as a likely reason for good data matching shown in Fig. 8. As a result, artificially made porous rocks reveal that such a relationship between sonic velocity and porosity can be controlled based on required circumstances.

### Chemical properties

**Figure 9:**
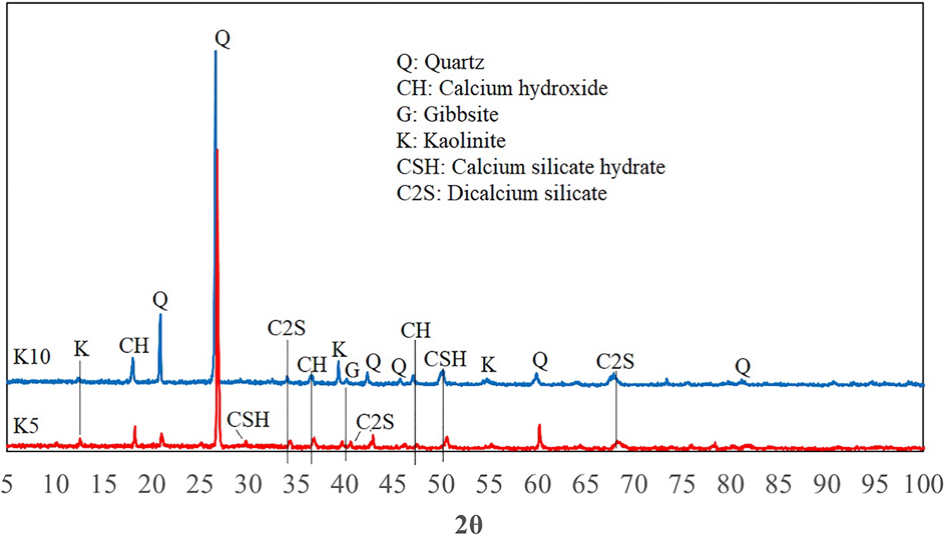
XRD analysis of a core sample containing kaolinite.

**Figure 10:**
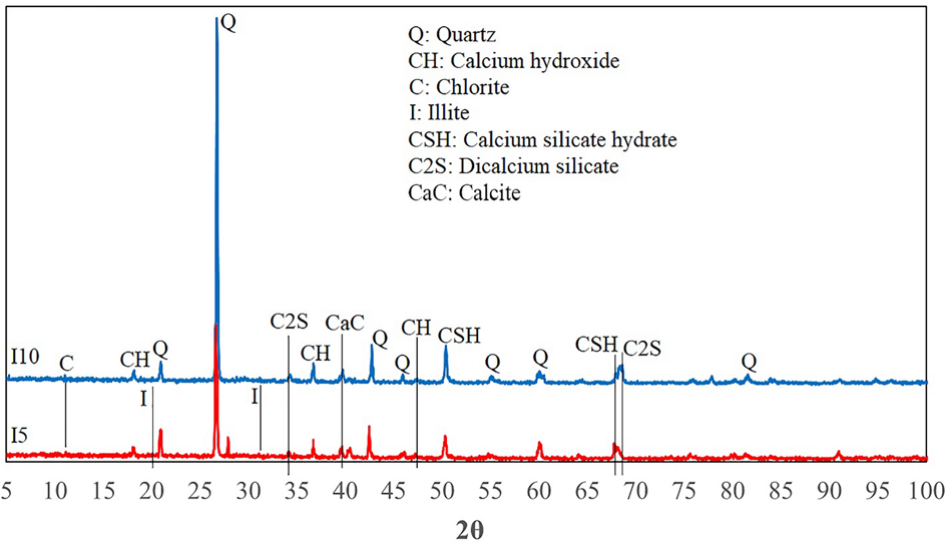
XRD analysis of a core sample containing illite.

**Figure 11:**
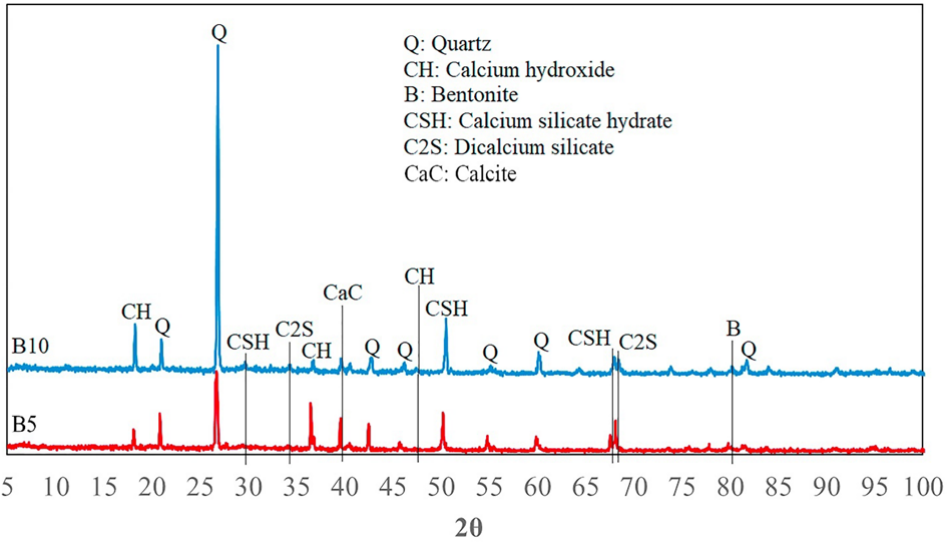
XRD analysis of a core sample containing bentonite.

**Figure 12:**
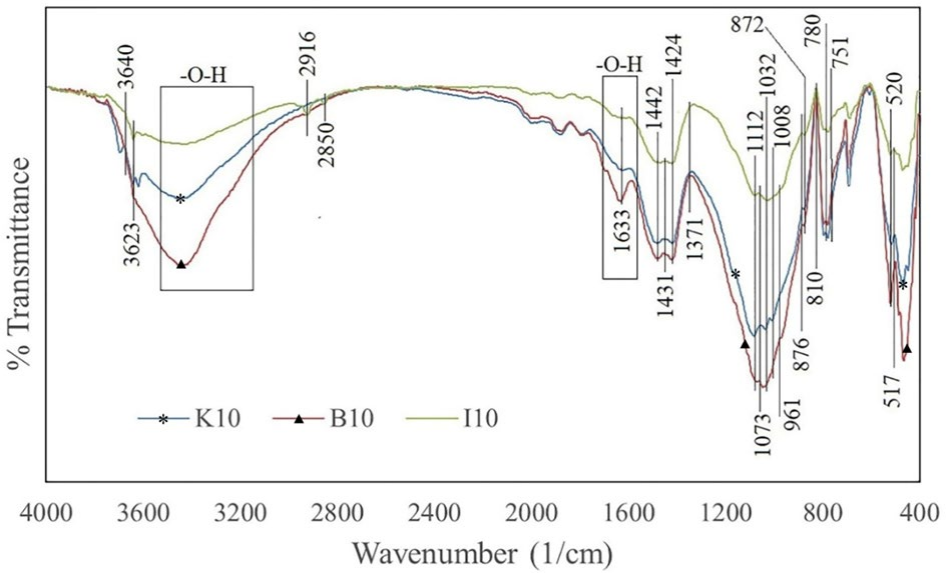
FTIR spectrum of K10, B10, and I10 samples.

X-ray diffraction (XRD) analysis is a non-destructive technique that provides detailed information about the crystallographic structure and chemical composition of a material^86^. XRD can be applied to detect chemical products and residual reactants resulting from chemical interactions between cement, clay materials, and water in the composition of rock samples. Thus, in addition to FESEM images, XRD analysis was performed on pulverized samples, which were obtained by crushing the samples using an agate mortar and pestle by an Inel Equinox 3000 X-Ray Diffractometer. The XRD traces were measured in operating conditions of 60 kV, 60 mA current, 0.0310 (2θ) step size, and 2° to 110° scanning range, and different mineral phases were quantified using peak intensity ratios. Moreover, using Fourier-transform infrared spectroscopy (FTIR), the mixture ingredients’ spectra were collected in the 4000–400 cm^−1^ mid-infrared spectral region at 0.4 cm^−1^ resolution. The results have been demonstrated in Figs. 9, 10, 11 and 12.

The crystal systems of each component formed in the samples are presented in Table 4.

**Table 4:** Crystal systems of chemical components.

Component	Crystal system
Quartz	Hexagonal
Dicalcium silicate	Monoclinic
Kaolinite	Anorthic
Gibbsite	Monoclinic
Calcium hydroxide (portlandite)	Hexagonal
Calcium carbonate	Rhombohedral
Calcium silicate	Hexagonal
Calcite	Hexagonal
Illite	Monoclinic
Chlorite	Monoclinic
Bentonite	Tetrahedra-octahedra-tetrahedra

The FTIR analysis can be used to define the chemical products formed in the reactions between Portland cement and clay minerals during the initial and ultimate curing processes. The main chemical reactions that may occur in the presence of clay minerals and Portland cement in an aqueous solution are presented in Table 5.

**Table 5:** Chemical reactions of clay minerals and Portland cement in an aqueous solution.

Reactant/product	Clay type	Chemical reaction	Reference
Tricalcium silicate	Kaolinite	$\underline{{{\text{2Ca}}_{{3}} {\text{SiO}}_{{5}} }} + {\text{7H}}_{{2}} {\text{O}} \to {\text{3CaO}} \cdot {\text{2SiO}}_{{2}} \cdot {\text{4H}}_{{2}} {\text{O}} + {\text{3Ca(OH)}}_{{2}}$	^87^
	Bentonite		
	Illite		
Dicalcium silicate	Kaolinite	$\underline{{{\text{2Ca}}_{{2}} {\text{SiO}}_{{4}} }} + {\text{5H}}_{{2}} {\text{O}} \to {\text{3CaO}} \cdot {\text{2SiO}}_{{2}} \cdot {\text{4H}}_{{2}} {\text{O}} + {\text{Ca(OH)}}_{{2}}$	^88^
	Bentonite		
	Illite		
Calcium silicate hydrate	Kaolinite	${\text{2Ca}}_{{3}} {\text{SiO}}_{{5}} + {\text{7H}}_{{2}} {\text{O}} \to \underline{{{\text{3CaO}} \cdot {\text{2SiO}}_{{2}} \cdot {\text{4H}}_{{2}} {\text{O}}}} + {\text{3Ca(OH)}}_{{2}}$	^15^
	Bentonite		
	Illite		
Calcium hydroxide (portlandite)	Kaolinite	${\text{2Ca}}_{{3}} {\text{SiO}}_{{5}} + {\text{7H}}_{{2}} {\text{O}} \to {\text{3CaO}} \cdot {\text{2SiO}}_{{2}} \cdot {\text{4H}}_{{2}} {\text{O}} + \underline{{{\text{3Ca(OH)}}_{{2}} }}$	^15^
	Bentonite		
	Illite		
Kaolinite	Kaolinite	$\underline{{{\text{Si}}_{{2}} {\text{O}}_{{5}} {\text{Al}}_{{2}} {\text{(OH)}}_{{4}} }} {\text{ + 6H}}^{ + } \to {\text{Al}}^{{3 + }} {\text{ + 2SiO}}_{{2}} {\text{ + 5H}}_{{2}} {\text{O}}$	^89^
Gibbsite	Kaolinite	$\underline{{{\text{Al(OH)}}_{{3}} }} {\text{ + 3H}}^{ + } \to {\text{Al}}^{{3 + }} {\text{ + 3H}}_{{2}} {\text{O}}$	^89^
Calcite	Illite	$\underline{{{\text{CaCO}}_{{3}} }} \to {\text{Ca}}^{{2 + }} {\text{ + CO}}_{{3}}^{{2 - }}$	^89^
	Bentonite		
Chlorite	Illite	$$\begin{gathered} {\text{15CaMg}}\left( {{\text{CO}}_{{3}} } \right)_{{2}} { + 2}\left[ {{\text{(K, Mg, Al)Si}}_{{2}} {\text{O}}_{{{10}}} {\text{(OH)}}_{{2}} } \right]{\text{ + 3SiO2 + 11H2O}} \to \hfill \\ {3}\left[ {\underline{{{\text{(Mg, Fe, Al)}}_{{6}} {\text{(Si, Al)6O}}_{{{10}}} {\text{(OH)}}_{{8}} }} } \right]{\text{ + 15CaCO}}_{{3}} {\text{ + 2K(OH) + 15CO}}_{{2}} \hfill \\ \end{gathered}$$	^90^
Calcium oxide	Kaolinite	$\underline{{{\text{CaO}}}} {\text{ + H}}_{{2}} {\text{O}} \to {\text{Ca(OH)}}_{{2}}$	^91^
	Bentonite		
	Illite		

Figures 9, 10 and 11 confirm the formation of the reaction products between cement and the clay minerals listed in Table 5. As can be seen from Figs. 9, 10 and 11, increasing the clay content can enhance the amount of chemical products. The reaction products can also be identified from the peaks in the FTIR analysis. In the FTIR test, the position of absorption peaks in the infrared region of the electromagnetic spectrum helps to identify a mineral's molecular bonds and building blocks based on the vibrations of molecular bonds of the minerals^92^. The FTIR spectrum of samples in Fig. 12 illustrates multiple absorption peaks related to kaolinite (1112 cm^−1^ is the longitudinal Si–O stretching mode, and the Si–O–Si and Al–O–Si stretching peaks of the tetrahedral layer were clearly seen at 1032, and 1008 cm^−1^, respectively)^93^. In addition, the FTIR spectra for bentonite (montmorillonite) show the absorption bands in 520, 751, 1633, 2916, 3623 cm^−1^^94–96^. The next four bands in the spectrum of 1633, 2850, 2916, and 3623 cm^−1^ are attributed to vibrations of illite^97^. The peaks at 517, 780, 810, and 1073 cm^−1^ could be expected for quartz^97–99^, and peaks at 876 and 1424 cm^-1^ for calcium hydroxide. The extensive band at 1424 cm^−1^ illustrates the Ca–O bond related to the carbonation of Ca(OH)_2_. Moreover, the strong bands between 3100 and 3400 cm^−1^ correspond to O–H stretching and bending vibrations, and the band at around 1625 cm^−1^ can be attributed to O–H vibrations, agrees the existence of the O–H bond in Ca(OH)_2_^100^. Moreover, calcite (CaCO_3_) has been documented to have peaks at 876 and 1431 cm^-1^^101^. The band at 961 cm^−1^ is related to a coupling of the vibrations of the Si–O–Si, Si–OH, and Si(OSi)_3_O–Ca groups, and the characteristic adsorptions at 875 cm^−1^ are related to Si–O bending vibration (calcium silicate hydrate (CSH))^102,103^. The water molecules and hydroxyl groups of the CSH-phases have a broad band in the 3100–3500 cm^−1^ region as well as an -OH bending mode between 1633 to 1663 cm^−1^^104^. The additional band can be marked in the FTIR spectra of hydrated Tricalcium silicate (C_3_S) in the range of 965, 1371, and 3640 cm^−1^^105^. In addition, the vibration for the bi-calcium silicate (C_2_S) develops at around 872 cm^−1^^106^. The results of the FTIR and XRD analyses reveal that the presence of clay minerals in the rock composition can lead to the formation of elements that are mentioned in Table 5 as factors influencing the rock properties.

### Geomechanical properties

**Figure 13:**
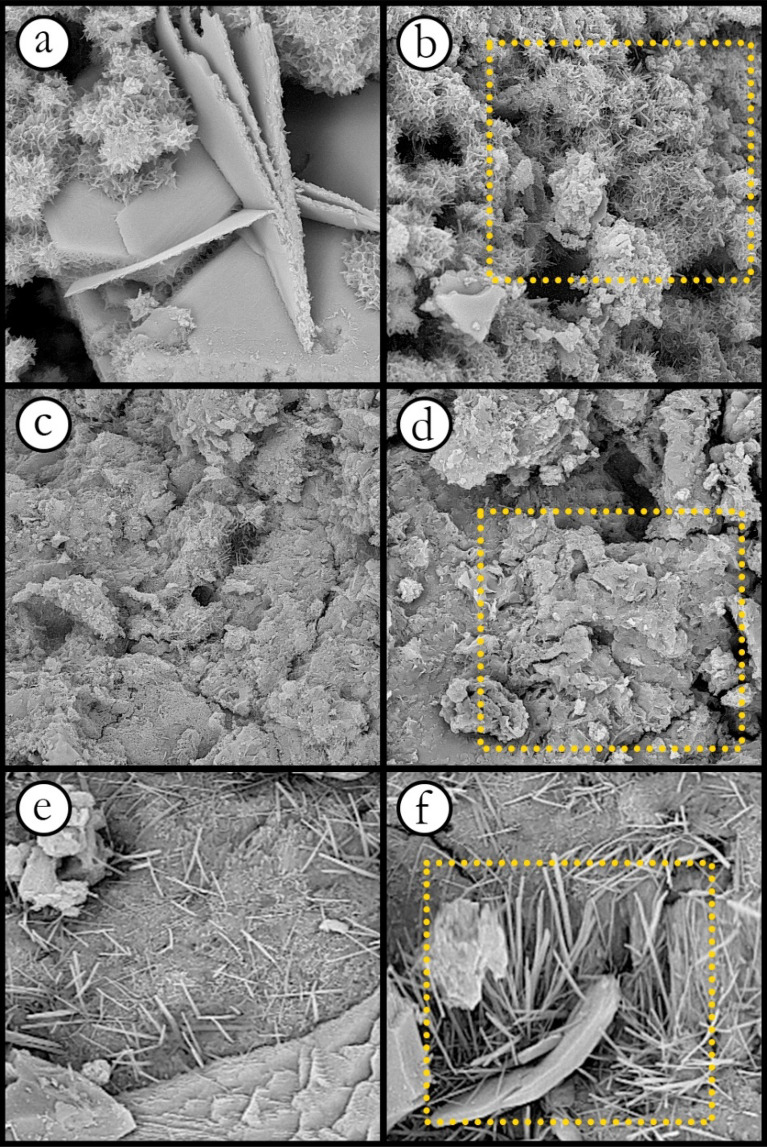
FESEM images of specimens, (**a**) kaolinite 5%, (**b**) kaolinite 10%, (**c**) bentonite 5%, (**d**) bentonite 10%, (**e**) illite 5%, and (**f**) illite 10% (view field: 28.9 µm).

A change in clay content can alter the rock structure and the form of the pore surfaces. To illustrate the effects of clay content on rock texture, a series of FESEM images were taken of samples with kaolinite, bentonite, and illite contents of 5% and 10%, and the results are shown in Fig. 13.

**Figure 14:**
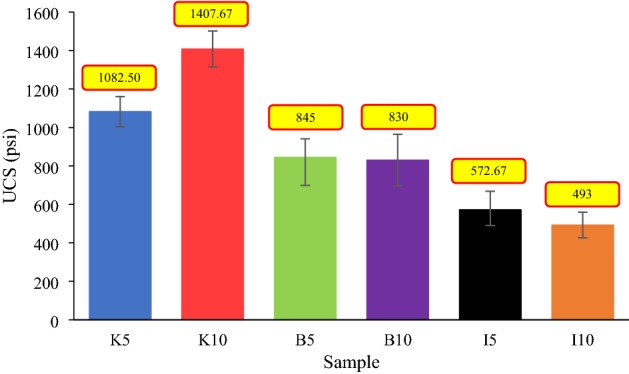
UCS test results of all samples.

**Figure 15:**
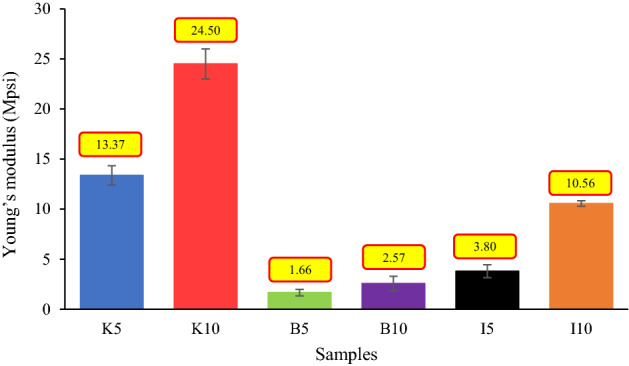
Young’s modulus of the specimens.

As highlighted in Fig. 13b,d and f, an increase in clay content (i.e. increasing the chemical products), which is one of the main factors in describing the rock characteristics, can remarkably change the structure of the samples. As a result, it would intensify adverse effects on the physical and geomechanical properties of the samples, such as porosity, permeability, UCS, and the Young’s modulus. In this study, several UCS tests, one of the most important and widely used geomechanical evaluation methods for estimating rock strength, were performed on all samples, and the results are shown in Figs. 14 and 15. It should be noted that for each material type, three samples were used for the UCS tests.

Rock porosity and cementation quality, as influential factors in modifying the UCS and the elastic modulus of a rock, can be affected by increasing the clay content. The UCS of a man-made porous rock sample depends inversely on its porosity and directly on the quality of cementation at the grain contacts^6,12,107^. In this regard, impurities in the cement at the grain boundaries can influence cement bond strength. Both the porosity reduction and increasing bond quality would result in an improvement in UCS. In general, a high clay content can lessen the rock's strength because the degree of cementation decreases, especially in the presence of water^108^. Besides, porosity reduction would lead to an increase in rock strength^6,12,15,109^. The results in Fig. 14 show that for samples containing kaolinite, a reduction in porosity remarkably improves UCS and can to some extent compensate for the detrimental effects of poor bond quality, as a 13% reduction in porosity results in a 30% improvement in UCS. On the contrary, the influence of poor cementation quality on the UCS of illite-containing specimens is much more important than the porosity. For instance, in samples containing illite, a decrease in porosity by 3.3% and a 5% increase in illite content would lead to a 14% reduction in UCS. The effects of the porosity reduction and the degree of bonding on the UCS of samples containing bentonite almost balance each other. To give a clear example, if the clay content of a sample increases by 5%, the porosity of the sample decreases by 4.1%, and consequently, the UCS diminishes by only 1.8%. Therefore, when it comes to changes in geomechanical characteristics, the predominant factor (i.e. a reduction in porosity or cementation quality) determines the ultimate impact of clay minerals on the properties of an engineered porous rock (increase or decrease). In other words, porosity and degree of cementation, which are related to the type and quantity of clay minerals, are the main controlling factors in such engineered porous samples affecting geomechanical characteristics.

The Young’s modulus data in Fig. 15 reveal that there is a direct relationship between clay content and elastic modulus. In this regard, the results indicated that samples containing illite had the greatest increase in Young’s modulus, while the smallest increase was observed in bentonite-containing specimens. Based on the results demonstrated in Fig. 15, a 5% increase in clay content would result in an 83%, 55%, and 178% improvement in the elastic modulus of samples containing kaolinite, bentonite, and illite, respectively. The Poisson's ratio data of the rock samples are given in Table 6.

**Table 6:** Poisson’s ratio of samples.

Sample	Clay type	Poisson’s ratio (–)
K5	Kaolinite	0.46
B5	Bentonite	0.18
I5	Illite	0.26
K10	Kaolinite	0.205
B10	Bentonite	0.25
I10	Illite	0.48

According to the data in Table 6, a higher proportion of kaolinite would lead to a sharp decrease in Poisson's ratio, while an increasing trend was observed for specimens containing bentonite and illite. Such waning and rising trends for kaolinite and bentonite, respectively, were reported by Mondol et al. (2015) in synthetic mudstones^110^. However, no clear relationship between clay content and Poisson’s ratio was observed.

**Figure 16:**
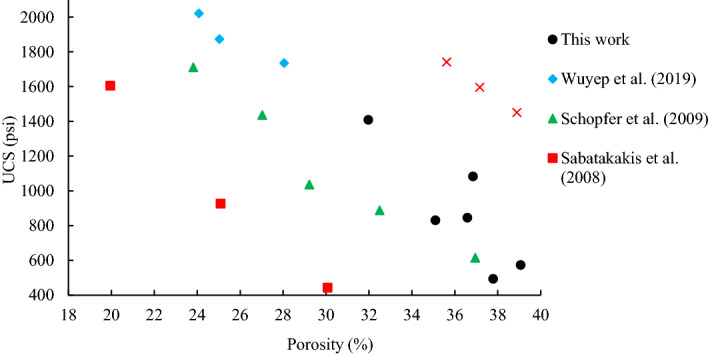
Comparison between UCS and porosity of the current research and the literature (data from^111–114^).

Figure 16 shows the comparison between the UCS data in terms of rock porosity of this study and a series of samples containing kaolinite, illite, and montmorillonite as clay minerals from the literature.

As can be seen from Fig. 16, the artificial specimens used in the current study can represent real specimens, thus the data obtained here could help researchers in their future studies.

## Conclusions

In this research study, a series of physico-chemical tests along with geomechanical tests were carried out on various sythetic samples to investigate the effects of type and content of clay minerals on rock properties. The following conclusions can be drawn from to the results obtained here:

- The results of the porosity and permeability tests showed that an increase in clay content by 5% (kaolinite, bentonite, and illite) reduces the porosity and permeability of the engineered rocks. Besides, the results revealed that changes in clay content can further affect permeability compared to porosity.

- FESEM images showed that kaolinite can lead to a fluffy shape in the material structure, while a needle form was observed in samples containing illite. Bentonite, due to its high specific surface area, can cover pore surfaces and create a coated surface. The fluffy form would block most flow paths and pore throats. As a result, kaolinite has a dramatic effect on porosity and permeability compared to bentonite and illite.

- Both compressive and shear sonic velocities in samples including various clay minerals show good exponential trends with the porosity data. It is likely due to the uniform and identical texture of the specimens. Accordingly, analogous samples can increase the accuracy of exponential correlations for estimating sonic velocities from porosity data.

- The results of the FTIR and XRD analyses reveal that an increase in clay content would greatly enhance the amount of chemical products formed in the reactions between cement and clay minerals during the initial and final curing processes.

- An increase in clay content may both reduce rock porosity and cementation quality as influential factors in changing the UCS and the elastic modulus of a rock. Either a porosity reduction or increasing the degree of cementation would lead to an improvement in UCS. The results indicated that samples containing illite experienced the largest increase in modulus of elasticity, while the smallest increase was observed in bentonite-containing specimens.

- For samples containing kaolinite, the reduction in porosity considerably improves UCS and compensates for the adverse effect of poor bond quality. As opposed to kaolinite, the influence of deterioration in cementation quality on the UCS of specimens containing illite is much greater than that of porosity. For bentonite- containing samples, the effects of decreasing porosity and low degree of cementation on UCS almost balance each other. Thus, when it comes to changes in geomechanical characteristics, the dominant factor determines the direction of the changes.

## Data Availability

The datasets used and/or analysed during the current study are available from the corresponding author on reasonable request.

## Abbreviations

UCS: Uniaxial compressive strength
PSD: Particle size distribution
wc/c: Water-clay/cement ratio
O: Octahedral
T: Tetrahedral
XRF: X-ray fluorescence spectrometry
LOI: Loss on ignition
K5: Kaolinite 5%
K10: Kaolinite 10%
B5: Bentonite 5%
B10: Bentonite 10%
I5: Illite 5%
I10: Illite 10%
XRD: X-ray diffraction
FTIR: Fourier-transform infrared spectroscopy
FESEM: Field emission scanning electron microscopy
wt%: Weight percent
